# Preliminary investigation of the association between air pollution exposure and childhood asthma hospitalizations from 2015 to 2018 in East China

**DOI:** 10.3389/fpubh.2025.1527214

**Published:** 2025-06-10

**Authors:** Yuling Bao, Jiawei Wang, Hui Huang, Zhe Sun, Mingyan Xue, Zilong Bian, Rui Jin, Qian Wu

**Affiliations:** ^1^Department of Respiratory, Children’s Hospital of Nanjing Medical University, Nanjing, China; ^2^College of Environmental Science and Engineering, Nankai University, Tianjin, China; ^3^Department of Biostatistics, School of Public Health, Nanjing Medical University, Nanjing, China; ^4^Department of Pediatrics, The First Affiliated Hospital, Nanjing Medical University, Nanjing, China; ^5^The Key Laboratory of Modern Toxicology of Ministry of Education and Department of Health Inspection and Quarantine, Nanjing Medical University, Nanjing, China

**Keywords:** air pollution, exposure, asthma, childhood, association

## Abstract

**Objectives:**

This study investigated whether exposure to air pollution remains a significant factor contributing to childhood asthma in China.

**Methods:**

Short-term exposure to air pollutants was assessed using daily average concentrations of pollutants at current and lag intervals (0–6 days) from 2015 to 2018. Long-term individual exposure in 2016 was estimated using land-use regression (LUR) models. The effects of short- and long-term exposure on childhood asthma hospitalizations were evaluated using generalized additive models and multiple time-dependent Cox regression models, respectively.

**Results:**

Hospitalizations for childhood asthma typically peaked in late spring and fall, with a higher prevalence of wheezing or asthma observed in male individuals than in female individuals. Hospital admissions were most frequent among children aged 0–3 years. However, no significant positive associations were observed between short- or long-term air pollutant exposure and daily childhood asthma hospitalizations, based on the applied statistical models and the levels of air pollution exposure measured during the study period.

**Conclusion:**

In this study, variability in air pollution exposure was not associated with variability in hospitalizations of children with asthma. Instead, asthma onset exhibited unique seasonal and demographic patterns.

## 1 Introduction

Over the last 30 years, China’s rapid economic growth and urbanization have exacerbated air pollution, leading to an increase in respiratory diseases (1). Asthma, in particular, has become a growing public health concern. According to the 2013 national survey conducted by the China Asthma Alliance, the overall prevalence of asthma in China was 1.24%. In cities such as Beijing and Shanghai, the prevalence increased by 147.9% and 190.2%, respectively, compared to a previous survey conducted a decade earlier (2). The rise in childhood asthma has been particularly alarming. The first national survey in 1990 reported a prevalence of 0.91% among children aged 0–14 years, which increased to 1.5% (3) by 2000 and reached 3.02% (4) in the 2010–2011 survey on childhood asthma and allergic diseases. Asthma is a multifactorial disease influenced by both genetic and environmental factors. Epidemiological studies have consistently linked air pollution exposure to increased prevalence and severity of asthma (5–8). For instance, one study evaluated the association between gaseous pollutants and emergency ambulance dispatches for asthma in southwestern China (9). However, some cohort studies in Europe and the United States have reported no significant association between air pollution and the prevalence of asthma (10, 11), highlighting the need for context-specific evaluations. Differences in ambient air pollution levels across regions may partly explain these discrepancies. According to China’s Environmental Status Bulletin (2014–2017), PM_2.5_ concentrations in the Yangtze River Delta decreased by 34.3% from 2013 to 2017, indicating an improvement in air quality. However, the beneficial effects of improved air quality on asthma have not been sufficiently investigated. Studies conducted in Taiwan have shown that improved air quality reduces the effect of PM_2.5_ on childhood asthma (12, 13). These findings emphasize the importance of considering regional pollution trends and population characteristics in assessing the relationship between air pollution and asthma. Recent evidence regarding the association between air pollution exposure and childhood asthma in China remains limited. This study aimed to assess whether air pollutants continue to significantly contribute to the increasing prevalence of asthma in children in China. Utilizing public data from 2015 to 2018, we analyzed the relationship between six common air pollutants (SO_2_, NO_2_, CO, O_3_, PM_2.5_, and PM_10_) and childhood asthma hospitalizations in Nanjing, located in the Yangtze River Delta region of East China.

## 2 Materials and methods

### 2.1 Data collection

Hospitalization data from 1 January 2015 to 31 December 2018 were collected from the Department of Respiratory Medicine at the Children’s Hospital of Nanjing Medical University (Hospital A) and Jiangsu Women and Children Health Hospital of Nanjing Medical University (Hospital B). This study included hospitalized cases with diagnoses of bronchial asthma (J45) and suspected asthma-acute asthmatic bronchitis (J21.901), based on the International Classification of Diseases, 10th revision. All cases were validated by a study physician.

Air pollution data, including daily concentrations of SO_2_, NO_2_, CO, O_3_-(8 h), PM_2.5_, and PM_10_, were obtained from the China National Environmental Monitoring Centre. Meteorological data, such as temperature, relative humidity, and wind speed, were obtained from the China Meteorological Administration and adjusted to account for the confounding effects of weather conditions.

### 2.2 Association between short-term exposure to air pollutants and childhood asthma hospitalizations using a generalized additive model

The relationship between air pollutants and meteorological variables was assessed using Spearman’s rank correlation coefficients. Time series analysis using a generalized additive model (GAM) was performed to evaluate the effects of each air pollutant (SO_2_, NO_2_, CO, O_3_, PM_2.5_, and PM_10_) on childhood asthma hospitalizations. Given that the hospitalization data followed an over-dispersed Poisson distribution, a quasi-Poisson regression was employed within the GAM framework.

To control for unmeasured confounders such as seasonal and long-term trends, the following adjustments were made: (1) natural cubic spline functions of calendar time with 5 degrees of freedom (df) per year, (2) natural smooth functions of the present-day temperature difference (3 df) and relative humidity (3 df) to exclude the confounding effects of weather, and (3) indicator variables for “day of the week” and “holidays.” The data were also stratified by sex, age group, and hospital level. A single-pollutant model was used to examine the effects of air pollution on asthma hospitalization, considering both single lag days (lag 0, 1, 2, 3, 4, 5, and 6) and multiple lag days (lag 0–1, 0–2, 0–3, 0–4, 0–5, and 0–6). The pollutant effect was quantified as the percentage change in daily asthma admissions per interquartile range increase, along with the corresponding 95% confidence interval (CI). Sensitivity analyses were conducted within subgroups. All analyses were performed using R software (version 3.5.3) with the mgcv package, and statistical significance was set at a *p*-value < 0.05.

### 2.3 Association between long-term exposure to air pollutants and childhood asthma hospitalizations using multiple time-dependent cox regression models

Individual exposures to air pollutants in 2016 were estimated based on residential locations using a spatiotemporal (ST) land-use regression (LUR) model for long-term exposure (details provided in Supplementary material). Data from nine monitoring stations in Nanjing in 2016 were used to measure the six air pollutants, with station details provided in Supplementary Table S1. The model’s independent variables included traffic, land use, meteorology, socioeconomic factors, and other relevant data, as detailed in Supplementary Table S2. Following standard LUR methodology, each independent variable was normalized before further analysis (Supplementary Table S3). The ST model was developed using the SpatioTemporal package (version 1.1.9) in R (version 3.5.1), with hyperparameters determined via cross-validation (Supplementary Table S4). The leave-one-out cross-validation method was used to assess the performance of the model. The coefficients of determination (R2cv) for SO_2_, NO_2_, CO, O_3_, PM_2.5_, and PM_10_, were 0.929, 0.876, 0.903, 0.959, 0.951, and 0.936, respectively. Subsequently, individual address coordinates replaced the monitoring sites, and air pollutant concentrations were predicted based on the address coordinates using buffer and distance parameters. Multiple time-dependent Cox regression models were used with asthma as the dependent variable, adjusting for age and sex. Time-dependent hazard ratios (HRs) and 95% CIs were estimated for air pollutants (SO_2_, NO_2_, CO, O_3_, PM_2.5_, and PM_10_) across all days in 2016. In addition, exposure was censored at the time of the first hospitalization, and a *p*-value of <0.05 was considered statistically significant.

## 3 Results

**Figure 1:**
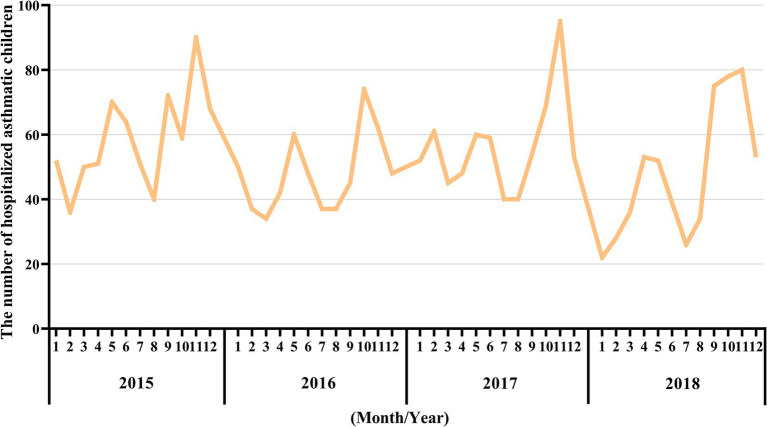
Monthly asthma-related hospital admissions during the study period (2015–2018). The X-axis represents the month and year, while the Y-axis represents the number of hospitalized asthmatic children in the corresponding month.

Nanjing, the provincial capital of Jiangsu Province, is located in the Yangtze River Delta region of China. Children’s Hospital of Nanjing Medical University and Jiangsu Women and Children Health Hospital of Nanjing Medical University are the two main pediatric hospitals in Nanjing. According to the Nanjing Hygiene Almanac (2015–2018), hospitalization data for children are reported only from these two hospitals. Table 1 shows the number of hospitalized cases of childhood asthma by age group and sex over the 4-year period. The cases were subdivided into three age groups (1–3) each year. The number of cases in age level 1 was significantly higher than that in the other two subgroups. On average, level 1 accounted for 77.5% of cases, compared to 16.7% in level 2 and 5.8% in level 3. The sex analysis revealed that, on average, 67.7% of asthma cases were male, indicating a sex difference in childhood asthma. Male children were significantly more likely to be sensitized to allergens (14, 15). This can be explained by differences in airway development and immune system function between male and female individuals (16, 17). Hospitalizations for childhood asthma peaked in late spring (March–May) and fall (September–November), as shown in Figure 1, consistent with the seasonal pattern of bronchial asthma exacerbations (18, 19).

**Table 1:** Summary of information about hospitalized childhood asthma cases (*N* = 2,529) and air pollutant indices in the Yangtze River Delta region during the study period (1 January 2015–31 December 2018).

Variables	2015 (*N* = 703)	2016 (*N* = 574)	2017 (*N* = 676)	2018 (*N* = 576)
Age level, *n* (%)				
Level 1 (0–3 years)	537 (76.4)	448 (78.0)	533 (78.8)	441 (76.6)
Level 2 (4–6 years)	119 (16.9)	98 (17.1)	105 (15.5)	100 (17.4)
Level 3 ( >6 years)	47 (6.7)	28 (4.9)	38 (5.6)	35 (6.1)
Gender, *n* (%)				
Female	213 (30.3)	189 (32.9)	221 (32.7)	192 (33.3)
Male	490 (69.7)	385 (67.1)	455 (67.3)	384 (66.7)
Air pollutant indice				
PM_2.5_ (μg/m^3^)	53	46	44	44
PM_10_ (μg/m^3^)	83	75	71	70
SO_2_ (μg/m^3^)	21	17	14	11
NO_2_ (μg/m^3^)	37	36	37	35
CO (mg/m^3^) (95%)	1.5	1.5	1.3	1.3
O_3_ (8h) (μg/m^3^) (90%)	163	159	170	167

The data of air pollutant indices are from Report on the State of the Environment in China (http://www.mee.gov.cn/).

**Figure 2:**
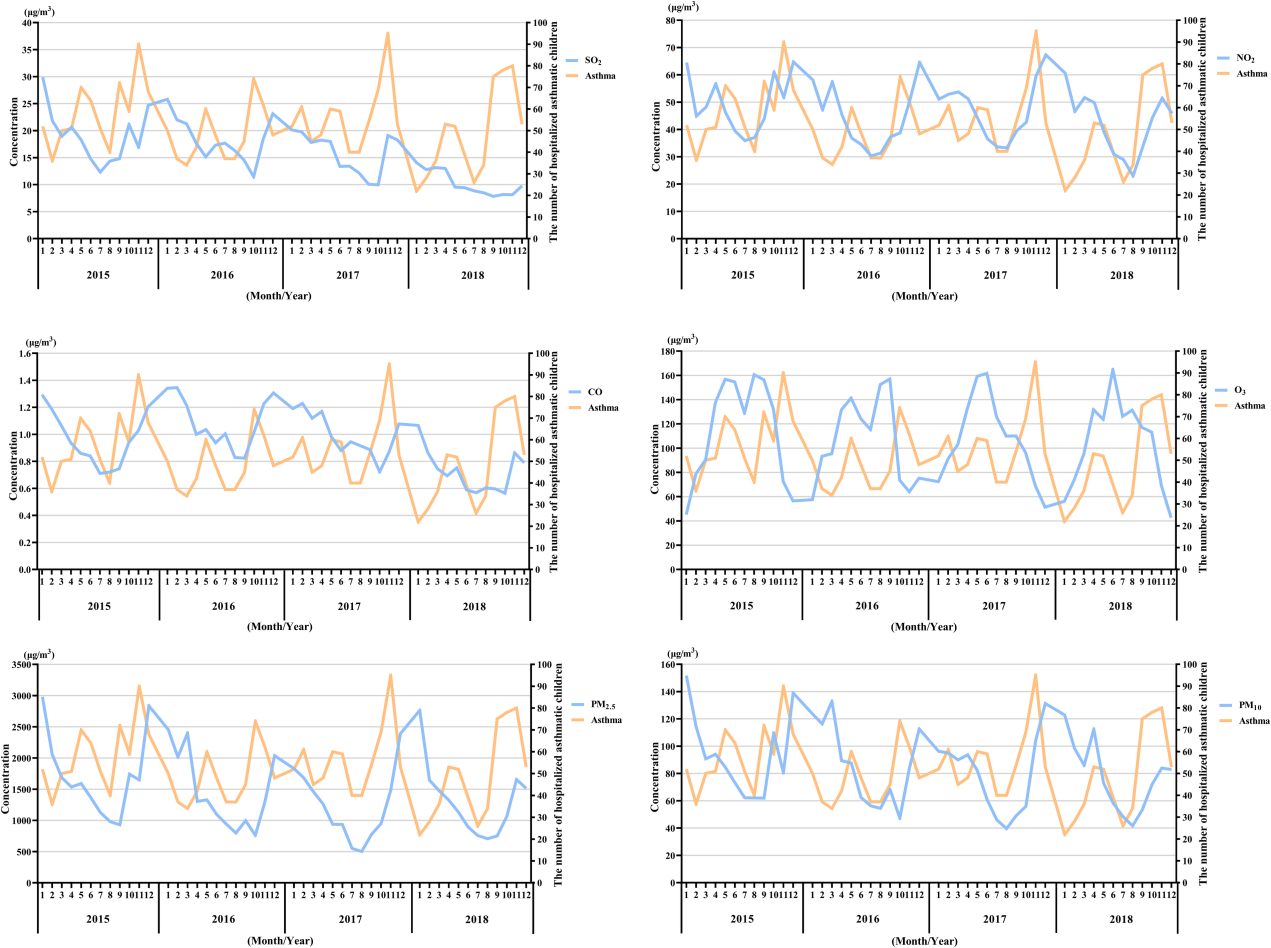
Pattern matching between the concentration trends of the six air pollutants and asthma-related hospital admissions during the study period (2015–2018). The X-axis represents the month and year, while the Y-axis on the left represents the monthly mean concentration of the pollutants, and the Y-axis on the right represents the number of hospitalized asthmatic children in the corresponding month.

**Figure 3:**
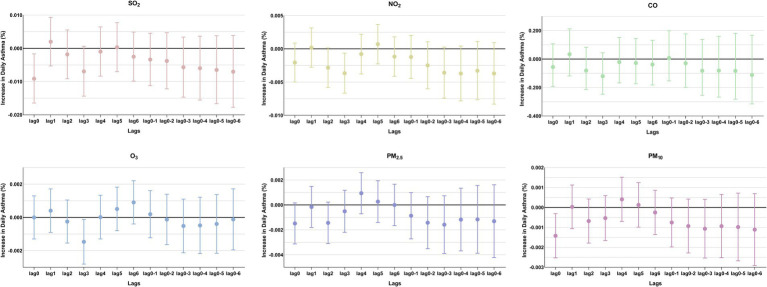
Percentage changes in daily asthma-related hospitalizations, with 95% confidence intervals, expressed as percentage deviations (%). In the single-pollutant model, an interquartile range (IQR) increase in the concentrations of SO_2_, NO_2_, CO, O_3_, PM_2.5_, and PM_10_ was associated with changes in asthma-related hospitalizations at different lag days.

**Figure 4:**
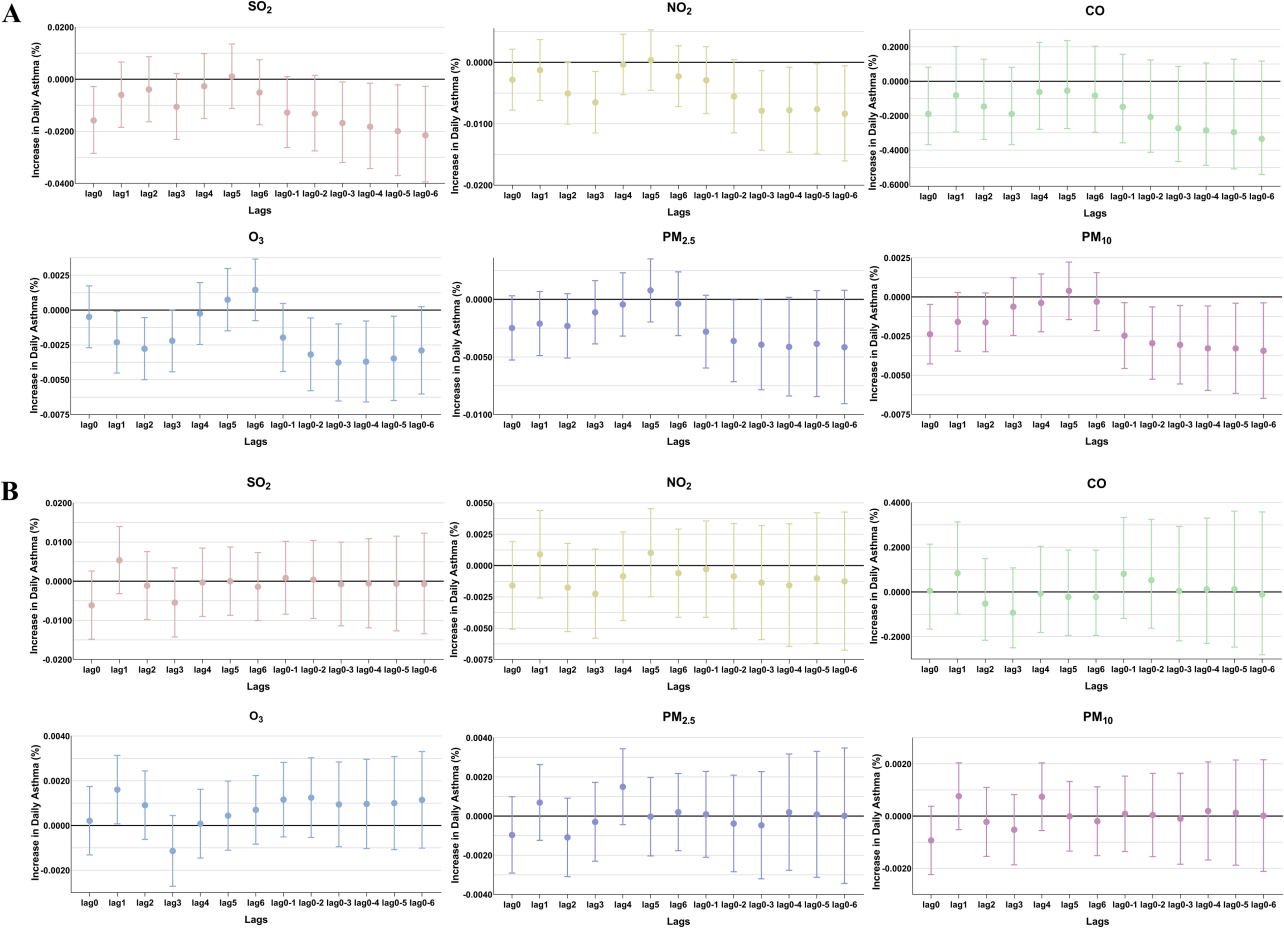
The patients were stratified by sex to analyze the association. Percent changes (95% CI) in daily asthma hospitalization deviations (%) stratified by sex: **(A)** female; **(B)** male.

**Figure 5:**
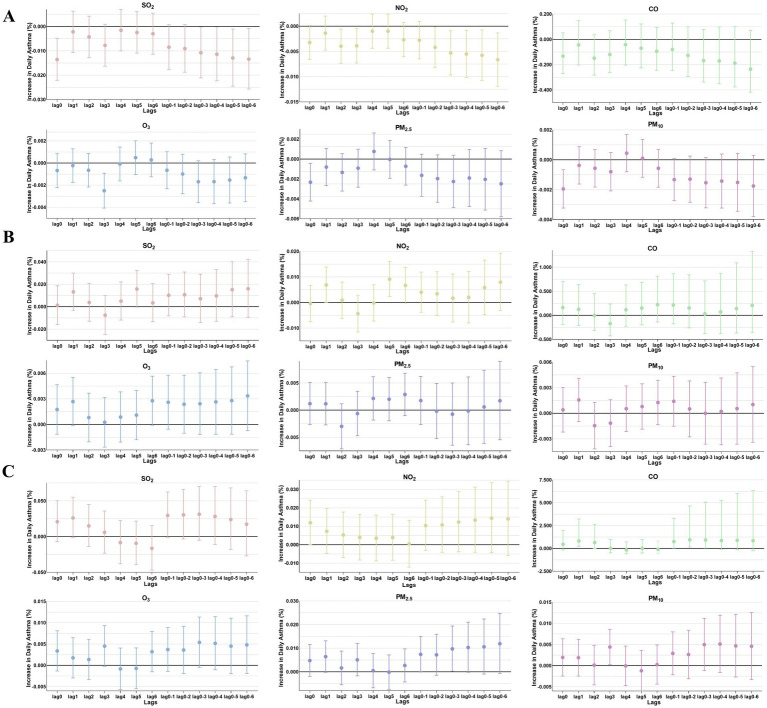
The patients were stratified by age to analyze the association. Percent changes (95% CI) in daily asthma hospitalization deviations (%) stratified by age: **(A)** 0–3 years; **(B)** 4–6 years; **(C)** >6 years.

**Figure 6:**
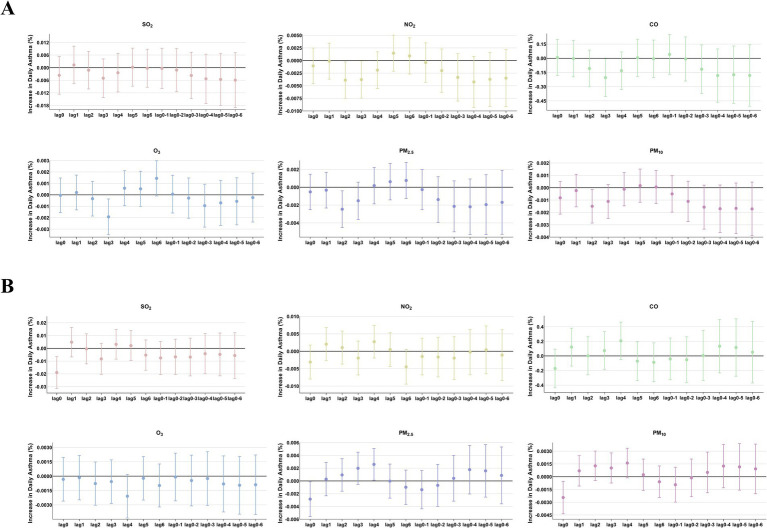
The patients were stratified by hospital level to analyze the association. Percent changes (95% CI) in daily asthma hospitalization deviations (%) stratified by hospital level: **(A)** Hospital A; **(B)** Hospital B.

**Figure 7:**
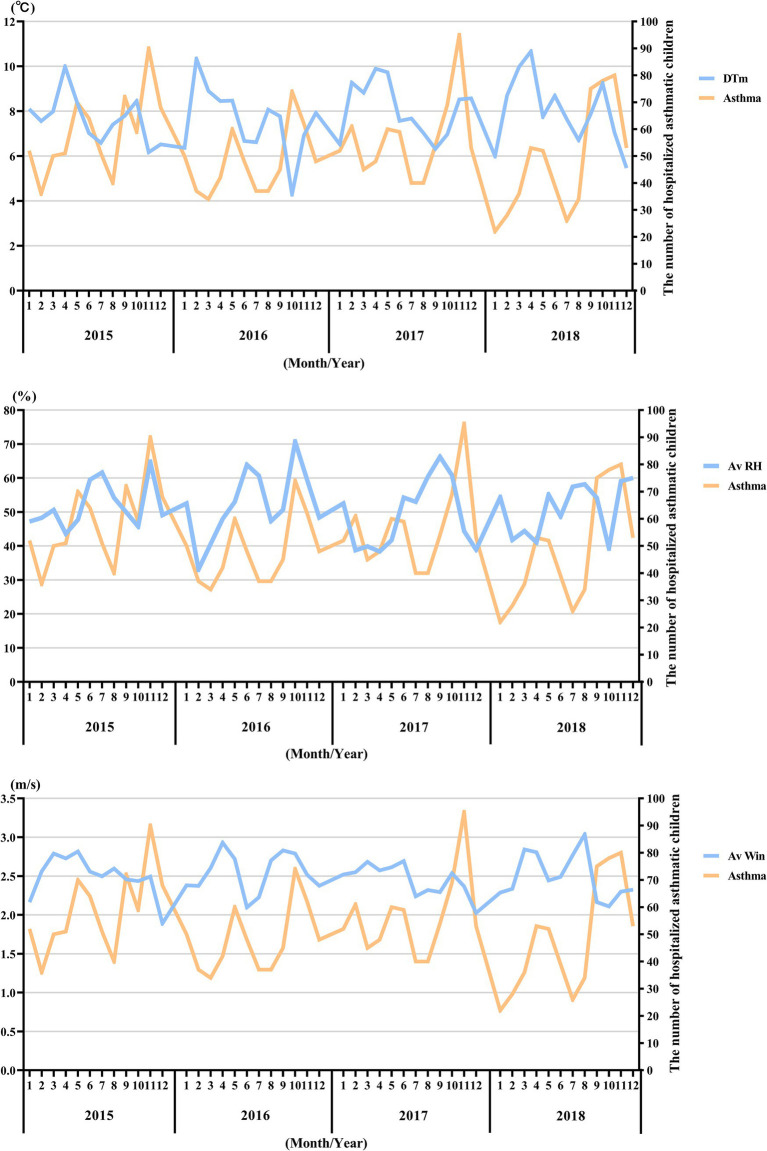
Pattern matching between temperature (°C), average relative humidity (%), average wind speed (m/s), and asthma-related hospital admissions during the study period (2015–2018). The X-axis represents the month and year, while the Y-axis on the left represents temperature (DTm, °C), average relative humidity (Av HR, %), and average wind speed (Av Win, m/s), respectively, and the Y-axis on the right represents the number of hospitalized asthmatic children in the corresponding month.

The comparison of the trend patterns between the monthly average concentrations of the six air pollutants and childhood asthma hospitalizations is shown in Figure 2. As shown, the concentrations of SO_2_, NO_2_, CO, PM_2.5_, and PM_10_ were usually higher from November to February of the following year. Unlike SO_2_, NO_2_, CO, PM_2.5_, and PM_10_, O_3_ concentrations were typically higher from May to September, with June, July, and August showing the highest ozone (O_3_) levels. By visual inspection, except for O_3_, the peaks in SO_2_, NO_2_, CO, PM_2.5_, and PM_10_ concentrations occurred after the peaks in asthma hospitalizations. Only the peaks in O_3_ concentrations preceded those in asthma hospitalizations, indicating a potential link to increased asthma-related hospital admissions. However, there was no significant positive association between the six common air pollutants and childhood asthma hospitalizations at the levels of air pollution exposure measured during the study period (Figure 3). Sex-stratified analysis revealed no significant association in both females (Figure 4A) and males (Figure 4B). Age-stratified analysis (0–3, 4–6, >6 years) showed no significant association across all subgroups (Figures 5A–C). However, as stratified by hospital level (Figures 6A, B), PM2.5 exposure at lag 4 day in Hospital B had a positive association with childhood asthmatic hospitalization. Monthly variations in temperature, average relative humidity, and average wind speed also aligned with hospital admissions, as shown in Figure 7, although the associations were not statistically significant.

Although the exposure estimates were derived from the LUR model, which offered improved accuracy, the results of the time-dependent Cox regression models indicated that SO_2_, NO_2_, CO, O_3_, PM_2.5_, and PM_10_ were not significant risk factors for asthma exacerbations requiring hospitalization, as shown in Table 2.

**Table 2:** Descriptive statistics from the time-dependent Cox regression models.

Air pollutant	Hazard ratios (HRs)	95% CI	*p* value
SO_2_	0.98	0.95, 1.02	0.389
NO_2_	1.01	0.98,1.05	0.566
CO	0.64	0.29, 1.42	0.270
O_3_	0.99	0.97, 1.01	0.308
PM_2.5_	1.01	0.97, 1.05	0.599
PM_10_	1.01	0.99, 1.03	0.271

## 4 Discussion

By the end of 2013, the majority of cities in China had established real-time monitoring for PM_2.5_ levels, and the new air quality standards were fully implemented nationwide in 2016. In the past 5 years, China has been implemented various policies to reduce fossil fuel combustion and vehicle exhaust emissions. As a result, significant reductions in PM_2.5_, PM_10_, and SO_2_ concentrations have been observed in the Yangtze River Delta, as shown in Table 1. While the positive effects of these policies on air quality are well-documented, their impact on childhood asthma remains insufficiently understood. In our study, we found that childhood asthma hospitalizations followed a seasonal pattern that did not entirely align with trends in air pollution. Pollen counts in Nanjing usually peak during April–May and September–October (20). The onset of spring leads to pollen allergies caused by grasses, weeds, and trees, while the transition to colder weather in the fall also exacerbates asthma attacks. The seasonal peaks in asthma hospitalizations observed in our study were consistent with these trends. However, we did not find a clear or significant positive association between the six air pollutants and childhood asthma hospitalizations from 2015 to 2018 in Nanjing (Supplementary Figure S1). However, the impact of O₃ on asthma prevalence varies compared to the effects of other pollutants such as SO_2_, NO_2_, CO, PM_2.5_, and PM_10_. Ozone acts as an oxidizing agent, which may induce respiratory tract inflammation (21, 22). It is also recognized as a secondary pollutant. Its formation is influenced by the presence of nitrogen oxides and photochemical transformation processes. Consequently, the exposure patterns of ozone can deviate substantially from those of other pollutants, such as nitrogen dioxide and fine particulate matter (23). In our study, during the observational years, ozone levels remained relatively high in the Yangtze River Delta region, partly due to the decrease in PM and increased solar radiation. This pattern is similar to the ozone pollution observed in the Pearl River Delta region (24), which may help explain the relationship between ozone concentrations and asthma outcomes. Although the associations between air pollutants and respiratory diseases have been widely studied, the results are complex and often inconclusive. Not all studies support a causal link between long-term exposure to air pollution and the prevalence of asthma (25). Indeed, etiological analyses from the International Study of Asthma and Allergies in Childhood (ISAAC) have revealed that the prevalence of asthma in less-polluted developed countries is generally much higher compared to those with higher levels of air pollution (26). In addition, two birth cohort studies found no association between traffic-related air pollution and atopic eczema, allergic sensitization, or bronchial hyperresponsiveness (27, 28), raising questions about the underlying mechanisms—whether irritation is caused by air pollution or allergic reactions due to allergens. Some studies have demonstrated that children residing in urban areas exhibit a higher susceptibility to asthma compared to those dwelling in rural or suburban regions. This elevated risk is, in part, attributed to their greater exposure to air pollution due to rapid urbanization and industrialization (29, 30). Nevertheless, cross-sectional studies carried out in Lanzhou, China, in 2017 and in Belgium have both revealed that the urban environment does not have a significant influence on children’s wheezing or asthma-related symptoms (31, 32). It is well known that children are susceptible to adverse effects of air pollution due to their developing lungs, immature immune systems, and metabolic pathways. In addition, even prenatal exposures might increase the postnatal risk of developing asthma later in life (33, 34). Reported associations in children have shown highly variable results. For example, a systematic review of cohort studies found that only prenatal exposure to NO_2_ (OR = 1.04, 95%CI: 1.01–1.07) and PM_10_ (1.08, 1.05–1.12) was associated with an increased risk of wheezing and asthma development in childhood. The effect was most pronounced in children aged 0–5 years, becoming weaker in older age groups. The researchers suggested that individual pollutant exposure assessments—such as LUR, inverse distance weighting (IDW), and personal monitors—used in these cohort studies might strengthen the observed positive associations between air pollution and wheezing and asthma (5). However, another systematic review published in the same year indicated that PM_10_ was not associated with asthma exacerbation in children. The researchers suggested that differences in methodologies for estimating air pollution concentrations across studies could lead to exposure misclassification (7). Alternatively, weaker associations have been observed in regions with lower levels of air pollution (35). Furthermore, the association between changes in ambient air pollution and incident asthma should be investigated in areas with predominantly low air pollution levels to avoid saturation effects.

In the present study, we raise the question of whether the role of air pollution in the prevalence of asthma has attenuated. It is also possible that the impacts of air pollution on asthma are overshadowed by other seasonal triggers that have a more substantial influence, such as bacterial, fungal, and viral infections. For example, recent studies have reported that air pollutants could significantly impact the city microbiome, which could further impact the prevalence of allergic diseases in a season-specific manner (36–38).

In summary, we believe that it is too arbitrary to draw a precise conclusion. In addition, even if air quality has improved significantly in recent years, the accumulated effects of environmental pollution still exist, preventing a decline in the prevalence of asthma. There are limitations in our study, such as the lack of a multicenter database evaluation and the use of single-year data for a long-term period, highlighting the need for further original studies to explore the relationship between air pollution exposure and childhood asthma. Nevertheless, a significant proportion of childhood asthma incidence/development may be attributable to air pollution (39–41). In the future, additional data should provide insights into the number of asthma cases that could potentially be prevented by reducing exposure to air pollution.

## 5 Conclusion

In our study, six nationally supervised air pollutant indices showed no clear or significant association with asthma hospitalizations in the studied population from 2015 to 2018 in East China, based on the levels of air pollution exposure measured during the study period. Our findings provide fundamental data and public health insights. The three national surveys on asthma prevalence among children aged 0–14 years in China were conducted in 1990, 2000, and 2010. These surveys indicated that the prevalence of asthma has continued to increase. A new round of national-level surveys on asthma prevalence is expected soon. As global emissions continue to rise, future research efforts should focus on identifying the pollutants most relevant to asthma, determining the most vulnerable children, and reducing exposure to improve child health.

## Data Availability

The original contributions presented in the study are included in the article/Supplementary material, further inquiries can be directed to the corresponding authors.

## References

[1] Hu LW Lawrence WR Liu Y Yang BY Zeng XW Chen W. Ambient air pollution and morbidity in Chinese. Adv Exp Med Biol. (2017) 1017:123–51. doi: 10.1007/978-981-10-5657-4_6, PMID: 29177961 PMC12846745

[2] National Cooperative Group on Childhood A, Institute of Environmental H, Related Product Safety CCfDC, Prevention, Chinese Center for Disease C, Prevention. Third nationwide survey of childhood asthma in urban areas of China. Zhonghua Er Ke Za Zhi. (2013) 51:729–35. 24406223

[3] Chen YZ National Cooperation Group On Childhood Asthma C. Comparative analysis of the state of asthma prevalence in children from two nation-wide surveys in 1990 and 2000 year. Zhonghua Jie He He Hu Xi Za Zhi. (2004) 27:112–6. 14990187

[4] Chen YZ National Cooperation Group On Childhood A. A nationwide survey in China on prevalence of asthma in urban children. Zhonghua Er Ke Za Zhi. (2003) 41:123–7. 14759318

[5] Hehua Z Qing C Shanyan G Qijun W Yuhong Z. The impact of prenatal exposure to air pollution on childhood wheezing and asthma: a systematic review. Environ Res. (2017) 159:519–30. doi: 10.1016/j.envres.2017.08.038, PMID: 28888196

[6] Khreis H Kelly C Tate J Parslow R Lucas K Nieuwenhuijsen M. Exposure to traffic-related air pollution and risk of development of childhood asthma: a systematic review and meta-analysis. Environ Int. (2017) 100:1–31. doi: 10.1016/j.envint.2016.11.012, PMID: 27881237

[7] Orellano P Quaranta N Reynoso J Balbi B Vasquez J. Effect of outdoor air pollution on asthma exacerbations in children and adults: systematic review and multilevel meta-analysis. PLoS One. (2017) 12:e0174050. doi: 10.1371/journal.pone.0174050, PMID: 28319180 PMC5358780

[8] Zhang Y Ni H Bai L Cheng Q Zhang H Wang S. The short-term association between air pollution and childhood asthma hospital admissions in urban areas of Hefei City in China: a time-series study. Environ Res. (2019) 169:510–6. doi: 10.1016/j.envres.2018.11.043, PMID: 30544078

[9] Chen J Jiang X Shi C Liu R Lu R Zhang L. Association between gaseous pollutants and emergency ambulance dispatches for asthma in Chengdu, China: a time-stratified case-crossover study. Environ Health Prev Med. (2019) 24:20. doi: 10.1186/s12199-019-0773-0, PMID: 30885130 PMC6421698

[10] Krefis AC Fischereit J Hoffmann P Pinnschmidt H Sorbe C Augustin M. Temporal analysis of determinants for respiratory emergency department visits in a large German hospital. BMJ Open Respir Res. (2018) 5:e000338. doi: 10.1136/bmjresp-2018-000338, PMID: 30487970 PMC6241969

[11] Annesi-Maesano I. It is not time to lower the guard! Eur Respir J. (2015) 45:589–91. doi: 10.1183/09031936.00008415, PMID: 25726533

[12] Chen BY Chen CH Chuang YC Wu YH Pan SC Guo YL. Changes in the relationship between childhood asthma and ambient air pollution in Taiwan: results from a nationwide survey repeated 5 years apart. Pediatr Allergy Immunol. (2019) 30:188–94. doi: 10.1111/pai.12999, PMID: 30371957

[13] Chen WY Lin CW Lee J Chen PS Tsai HJ Wang JY. Decreasing ten-year (2008-2018) trends of the prevalence of childhood asthma and air pollution in southern Taiwan. World Allergy Organ J. (2021) 14:100538. doi: 10.1016/j.waojou.2021.100538, PMID: 34025904 PMC8102795

[14] Mandhane PJ Greene JM Cowan JO Taylor DR Sears MR. Sex differences in factors associated with childhood- and adolescent-onset wheeze. Am J Respir Crit Care Med. (2005) 172:45–54. doi: 10.1164/rccm.200412-1738OC, PMID: 15805179 PMC2718447

[15] Almqvist C Worm M Leynaert B working group of GALENWPG. Impact of gender on asthma in childhood and adolescence: a GA2LEN review. Allergy. (2008) 63:47–57. doi: 10.1111/j.1398-9995.2007.01524.x, PMID: 17822448

[16] Chaves GS Freitas DA Santino TA Nogueira PAM Fregonezi GA Mendonca KM. Chest physiotherapy for pneumonia in children. Cochrane Database Syst Rev. (2019) 1:CD010277. doi: 10.1002/14651858.CD010277.pub3, PMID: 30601584 PMC6353233

[17] Timmons BW Tarnopolsky MA Snider DP Bar-Or O. Immunological changes in response to exercise: influence of age, puberty, and gender. Med Sci Sports Exerc. (2006) 38:293–304. doi: 10.1249/01.mss.0000183479.90501.a0, PMID: 16531898

[18] Eder W Ege MJ von Mutius E. The asthma epidemic. N Engl J Med. (2006) 355:2226–35. doi: 10.1056/NEJMra054308, PMID: 17124020

[19] Gerhardsson de Verdier M Gustafson P McCrae C Edsbacker S Johnston N. Seasonal and geographic variations in the incidence of asthma exacerbations in the United States. J Asthma. (2017) 54:818–24. doi: 10.1080/02770903.2016.1277538, PMID: 28102717

[20] Fang Y Ma C Bunting MJ Ding A Lu H Sun W. Airborne pollen concentration in Nanjing, eastern China, and its relationship with meteorological factors. J Geophys Res Atmos. (2018) 123:9026. doi: 10.1029/2018JD029026, PMID: 39712630

[21] Michaudel C Mackowiak C Maillet I Fauconnier L Akdis CA Sokolowska M. Ozone exposure induces respiratory barrier biphasic injury and inflammation controlled by IL-33. J Allergy Clin Immunol. (2018) 142:942–58. doi: 10.1016/j.jaci.2017.11.044, PMID: 29331644

[22] Wiegman CH Li F Ryffel B Togbe D Chung KF. Oxidative stress in ozone-induced chronic lung inflammation and emphysema: a facet of chronic obstructive pulmonary disease. Front Immunol. (2020) 11:1957. doi: 10.3389/fimmu.2020.01957, PMID: 32983127 PMC7492639

[23] Simpson DAA Mills G Solberg S Uddling J. Ozone — the persistent menace: interactions with the N cycle and climate change. Curr Opin Environ Sustain. (2014):9. doi: 10.1016/j.cosust.2014.07.008

[24] Zhao M Zhang Y Pei C Chen T Mu J Liu Y. Worsening ozone air pollution with reduced NO(x) and VOCs in the Pearl River Delta region in autumn 2019: implications for national control policy in China. J Environ Manag. (2022) 324:116327. doi: 10.1016/j.jenvman.2022.116327, PMID: 36183531

[25] Anderson HR Favarato G Atkinson RW. Long-term exposure to outdoor air pollution and the prevalence of asthma: meta-analysis of multi-community prevalence studies. Air Qual Atmos Health. (2013) 6:57–68. doi: 10.1007/s11869-011-0145-4

[26] Asher MI Weiland SK. The international study of asthma and allergies in childhood (ISAAC). Clin Exp Allergy. (1998) 28:52–66. doi: 10.1046/j.1365-2222.1998.028s5052.x 9988448

[27] Gehring U Wijga AH Brauer M Fischer P de Jongste JC Kerkhof M. Traffic-related air pollution and the development of asthma and allergies during the first 8 years of life. Am J Respir Crit Care Med. (2010) 181:596–603. doi: 10.1164/rccm.200906-0858OC, PMID: 19965811

[28] McConnell R Islam T Shankardass K Jerrett M Lurmann F Gilliland F. Childhood incident asthma and traffic-related air pollution at home and school. Environ Health Perspect. (2010) 118:1021–6. doi: 10.1289/ehp.0901232, PMID: 20371422 PMC2920902

[29] Priftis KN Mantzouranis EC Anthracopoulos MB. Asthma symptoms and airway narrowing in children growing up in an urban versus rural environment. J Asthma. (2009) 46:244–51. doi: 10.1080/02770900802647516, PMID: 19373631

[30] Liu P Wang X Fan J Xiao W Wang Y. Effects of air pollution on hospital emergency room visits for respiratory diseases: urban-suburban differences in eastern China. Int J Environ Res Public Health. (2016) 13:341. doi: 10.3390/ijerph13030341, PMID: 27007384 PMC4809004

[31] Cao S Wen D Li S Duan X Zhang Y Gong J. Changes in children’s asthma prevalence over two decades in Lanzhou: effects of socioeconomic, parental and household factors. J Thorac Dis. (2020) 12:6365–78. doi: 10.21037/jtd-19-crh-aq-008, PMID: 33209475 PMC7656413

[32] Wieringa MH Vermeire PA Van Bever HP Nelen VJ Weyler JJ. Higher occurrence of asthma-related symptoms in an urban than a suburban area in adults, but not in children. Eur Respir J. (2001) 17:422–7. doi: 10.1183/09031936.01.17304220, PMID: 11405520

[33] Chung HW Hsieh HM Lee CH Lin YC Tsao YH Wu HW. Prenatal and postnatal exposure to ambient air pollution and preschool asthma in neonatal jaundice infants. J Inflamm Res. (2022) 15:3771–81. doi: 10.2147/JIR.S366336, PMID: 35832831 PMC9271683

[34] Lu C Zhang Y Li B Zhao Z Huang C Zhang X. Interaction effect of prenatal and postnatal exposure to ambient air pollution and temperature on childhood asthma. Environ Int. (2022) 167:107456. doi: 10.1016/j.envint.2022.107456, PMID: 35952466

[35] Kuo CY Chan CK Huang JL Wu CY Phan DV Lo HY. Decline in hospitalization for childhood asthma in different air pollution regions in Taiwan, 2001-2012. Int J Environ Health Res. (2022) 32:95–105. doi: 10.1080/09603123.2020.1729964, PMID: 32073299

[36] Chen Y Fu X Ou Z Li J Lin S Wu Y. Environmental determinants and demographic influences on global urban microbiomes, antimicrobial resistance and pathogenicity. NPJ Biofilms Microbiomes. (2023) 9:94. doi: 10.1038/s41522-023-00459-4, PMID: 38062054 PMC10703778

[37] Sun Y Meng Y Ou Z Li Y Zhang M Chen Y. Indoor microbiome, air pollutants and asthma, rhinitis and eczema in preschool children—a repeated cross-sectional study. Environ Int. (2022) 161:107137. doi: 10.1016/j.envint.2022.107137, PMID: 35168186

[38] McCauley KE Flynn K Calatroni A DiMassa V LaMere B Fadrosh DW. Seasonal airway microbiome and transcriptome interactions promote childhood asthma exacerbations. J Allergy Clin Immunol. (2022) 150:204–13. doi: 10.1016/j.jaci.2022.01.020, PMID: 35149044

[39] Khreis H Cirach M Mueller N de Hoogh K Hoek G Nieuwenhuijsen MJ. Outdoor air pollution and the burden of childhood asthma across Europe. Eur Respir J. (2019) 54:1802194. doi: 10.1183/13993003.02194-2018, PMID: 31391220

[40] Khreis H de Hoogh K Nieuwenhuijsen MJ. Full-chain health impact assessment of traffic-related air pollution and childhood asthma. Environ Int. (2018) 114:365–75. doi: 10.1016/j.envint.2018.03.008, PMID: 29602620

[41] Khreis H Ramani T de Hoogh K Mueller N Rojas-Rueda D Zietsman J. Traffic-related air pollution and the local burden of childhood asthma in Bradford, UK. Int J Transp Sci Technol. (2019) 8:116–28. doi: 10.1016/j.ijtst.2018.07.003

