# Supplementary material for: Preliminary investigation of the association between air pollution exposure and childhood asthma hospitalizations from 2015 to 2018 in East China
Source: Front Public Health. 2025 Jun 10;13:1527214. doi: 10.3389/fpubh.2025.1527214 (PMC12185405; doi:10.3389/fpubh.2025.1527214)
Supplement: Supplementary file 1 [file Supplementary_file_1.docx]

# Preliminary investigation of the association between air pollution exposure and childhood asthma from 2015 to 2018 in East China

Yuling Bao^a^, Jiawei Wang^b^, Hui Huang^c^, Zhe Sun^c^, Mingyan Xue^c^, Zilong Bian^c^, Rui Jin^d*^, Qian Wu^e*^

^a^ Department of Respiratory, Children’s Hospital of Nanjing Medical University, Nanjing 210008, China.

^b^ College of Environmental Science and Engineering, Nankai University, Tianjin, 300350, China.

^c^ Department of Biostatistics, School of Public Health, Nanjing Medical University, Nanjing 211166, China.

^d^ Department of Pediatrics, the First Affiliated Hospital, Nanjing Medical University, Nanjing 210029, China.

^e^ The Key Laboratory of Modern Toxicology of Ministry of Education and Department of Health Inspection and Quarantine, Nanjing Medical University, Nanjing 211166, China.

*Corresponding authors: Qian Wu: [wuqian@njmu.edu.cn](mailto:wuqian@njmu.edu.cn); Rui Jin: [jr8622@163.com](mailto:jr8622@163.com)

**LUR methods**

Individual exposures to air pollutants in 2016 were estimated at residential location from spatiotemporal (ST) land use regression model for long-term exposure. Briefly, six kinds of air pollutants monitoring data of nine monitoring stations in Nanjing in 2016 were used, and the information of monitoring stations were shown in **Table S1**. The independent variables of modeling involve traffic, land use, meteorology, social economy and other variables, and the data sources were shown in **Table S2** similar to previous LUR studies, each independent variable was normalized before further analysis as shown in **Table S3**. We used SpatioTemporal package version1.1.9 in R.3.5.1 to establish ST model, and hyperparameters were determined by cross-validation as shown in **Table S4**. The leave-one-out cross validation method was used to assess the model performance. The coefficient of determination R^2^cv of CO, NO_2_, O_3_, PM_10_, PM_2.5_, and SO_2_ was 0.903, 0.876, 0.959, 0.936, 0.951 and 0.929. After that，we replaced previous monitoring site with individual’s address coordinate，compute the parameters including the buffer of independent variables and the shortest distanced and predicted the air pollutant concentration of the time panels according to the address coordinate.

# Table S1 Information of air pollutant monitoring sites in Nanjing in the year of 2016.

| ID | NAME | Longitude | Latitude |
| --- | --- | --- | --- |
| 1 | Maigao Bridge | 118.803 | 32.1083 |
| 2 | Caochang Gate | 118.749 | 32.0572 |
| 3 | Shanxi Road | 118.778 | 32.0723 |
| 4 | Zhonghua Gate | 118.777 | 32.0144 |
| 5 | Ruijin Road | 118.803 | 32.0314 |
| 6 | Xuanwu Lake | 118.795 | 32.0775 |
| 7 | Pukou | 118.626 | 32.0878 |
| 8 | Olympic Sports Center. | 118.737 | 32.0092 |
| 9 | Xianlin University Town | 118.907 | 32.105 |

# Table S2 Description of the data sources.

| Data | Description | Source | Spatial^a^ | Temporal^b^ |
| --- | --- | --- | --- | --- |
| Ambient air quality | Hourly CO; NO2; O3; PM10; MP2.5; SO2 Concentration | China National Environmental Monitoring Center (2016). (http://106.37.208.233:20035/) | N/A | Hour |
| Meteorology | Temperature, Humidity, Wind speed, Wind direction, pressure per hour | Nanjin Meteorological Bureau (2016). (http://js.cma.gov.cn/dsjwz/njs/) | N/A | Hour |
|  | Net radiation per day | NASA EARTH OBSERVATIONS (2016). (https://neo.sci.gsfc.nasa.gov/view.php?datasetId=CERES_NETFLUX_D&year=2016) | 0.25^。^ | Day |
| Road map | Digital network of railways and road | Open Street Map (2016).  (https://ww w.openstreetmap.org) | N/A | N/A |
| Land use | Forest, Grassland, Farmland, Urban, Rural settlement, Construction land, Unused land, Water | FROM-GLC10. (http://data.ess.tsinghua.edu.cn/fromglc10_2017v01.html) | 10m | N/A |
| Elevation | Digital elevation model (DEM) | U.S. Geological Survey (GDEMV2). (http://glovis.usgs.gov/) | 30m | N/A |
| Population Density | Resident population of each grid | Chinese Statistics Bureau (2015). (http://www.stats.gov.cn/tjsj/pcsj/) | 100m | N/A |
| Gross domestic product (GDP) | Yearly GDP of each grid | Chinese Statistics Bureau (2010). (http://www.stats.gov.cn/tjsj/pcsj/) | 1km | N/A |
| Normalized Difference Vegetation Index (NDVI) values | NDVI for per 16 days | Level-1 and Atmosphere Archive & Distribution System Distributed Active Archive Center (2016).  (http://ladsweb.nascom.nasa.gov/) | 250m | 16Day |
| aerosol optical thickness (AOD) |  | The Atmospheric Archive and Distribution System (2016). (http://ladsweb.nascom.nasa.gov/data/search.html) | 1km | 1Day |

^a^ spatial resolution of data

^b^ temporal resolution of data

# Table S3 Response and Explanatory variables for case study.

| **Variable** | **Buffer radii**^a^ | **Unit** |
| --- | --- | --- |
| **Response Variable** |  |  |
| Daily mean Carbon monoxide concentration | N/A | mg |
| Daily mean Nitrogen dioxide concentration | N/A | μg |
| Daily 8-h moving average ozone concentration | N/A | μg |
| Daily mean PM10 concentration | N/A | μg |
| Daily mean PM2.5 concentration | N/A | μg |
| Daily mean Sulfur dioxide concentration | N/A | μg |
| **Category: Traffic** |  |  |
| Total bus station numbers | 25m, 50m, 100m, 150m, 300m, 400m, 500m, 750m, 1km | N |
| Total level-1 road length | 25m, 50m, 100m, 150m, 300m, 400m, 500m, 750m, 1km | m |
| Total level-2 road length | 25m, 50m, 100m, 150m, 300m, 400m, 500m, 750m, 1km | m |
| Total level-3 road length | 25m, 50m, 100m, 150m, 300m, 400m, 500m, 750m, 1km | m |
| Total level-4 road length | 25m, 50m, 100m, 150m, 300m, 400m, 500m, 750m, 1km | m |
| Total bus route length | 25m, 50m, 100m, 150m, 300m, 400m, 500m, 750m, 1km | m |
| Total railway length | 25m, 50m, 100m, 150m, 300m, 400m, 500m, 750m, 1km | m |
| Distance to nearest level-1 road | N/A | m |
| Distance to nearest level-2 road | N/A | m |
| Distance to nearest level-3 road | N/A | m |
| Distance to nearest level-3 road | N/A | m |
| Distance to bus route | N/A | m |
| Distance to railway | N/A | m |
| **Category: Economic and Census Variables** |  |  |
| Population Density | 100m, 300m, 500m, 750m, 1km, 2km, 3km, 4km, 5km | N |
| Gross domestic product | 1km, 1.5km, 2km, 2.5km, 3km, 4km, 5km | ten thousand dollars |
| **Category: Land cover** |  |  |
| Area of land use category: Water body | 50m, 100m, 150m, 300m, 400m, 500m, 750m, 1km, 1.5km, 2km, 2.5km, 3km, 4km, 5km | m^2^ |
| Area of land use category: Grassland | 50m, 100m, 150m, 300m, 400m, 500m, 750m, 1km, 1.5km, 2km, 2.5km, 3km, 4km, 5km | m^2^ |
| Area of land use category: Urban | 50m, 100m, 150m, 300m, 400m, 500m, 750m, 1km, 1.5km, 2km, 2.5km, 3km, 4km, 5km | m^2^ |
| Area of land use category: Forest | 50m, 100m, 150m, 300m, 400m, 500m, 750m, 1km, 1.5km, 2km, 2.5km, 3km, 4km, 5km | m^2^ |
| Area of land use category: Rural settlement | 50m, 100m, 150m, 300m, 400m, 500m, 750m, 1km, 1.5km, 2km, 2.5km, 3km, 4km, 5km | m^2^ |
| Area of land use category: Farmland | 50m, 100m, 150m, 300m, 400m, 500m, 750m, 1km, 1.5km, 2km, 2.5km, 3km, 4km, 5km | m^2^ |
| Area of land use category: Construction land | 50m, 100m, 150m, 300m, 400m, 500m, 750m, 1km, 1.5km, 2km, 2.5km, 3km, 4km, 5km | m^2^ |
| Area of Land Use category: Unused land | 50m, 100m, 150m, 300m, 400m, 500m, 750m, 1km, 1.5km, 2km, 2.5km, 3km, 4km, 5km | m^2^ |
| Area of buildings | 50m, 100m, 150m, 300m, 400m, 500m, 750m, 1km, 1.5km, 2km, 2.5km, 3km, 4km, 5km | m^2^ |
| Count of buildings | 50m, 100m, 150m, 300m, 400m, 500m, 750m, 1km, 1.5km, 2km, 2.5km, 3km, 4km, 5km | N |
| Distance to water | N/A | m |
| Normalized Difference Vegetation Index (NDVI) values | 250m, 500m, 1km, 2.5km, 5km, 7.5km, 10km | N/A |
| Elevation | N/A | m |
| **Category: Meteorological Data** |  |  |
| Net radiation | N/A |  |
| Daily average velocity of wind, Daily maximum velocity of wind^c^ | N/A | m/s |
| Daily average direction of wind^c^ | N/A | N/A |
| Daily average temperature, Daily maximum temperature, Daily average temperature between 9 am to 9 pm | N/A | Celsius degree |
| Daily average relative humidity, Daily average relative humidity between between 9 am to 9 pm | N/A | N/A |
| Daily average atmospheric pressure | N/A | hPa |
| Daily average precipitation | N/A | mm |
| Aerosol optical thickness | N/A | N/A |
| **Category: Time and Geographical position** |  |  |
| Longitude, Latitude | N/A | degree |
| Date | N/A | N/A |

^a^ Measures were calculated within buffers with different radii.

^b^ n represents the different buffer radii

^c^ Statistical results of wind vector

# Table S4 The hyperparameters of the Spatiotemporal models.

| Pollutants | No. of  temporal  trends | Df | No. of PLS scores | | Spatial smoothing | |
| --- | --- | --- | --- | --- | --- | --- |
|  |  |  | LTM (β0) | Temporal  trends (βi) | LTM (β0) | Temporal  trends (βi) |
| CO | 2 | 100 | 3 | 4,2 | Yes | Yes |
| NO_2_ | 2 | 100 | 4 | 3 | Yes | Yes |
| O_3__8h | 2 | 100 | 3 | 4,4 | Yes | Yes |
| PM_10_ | 1 | 100 | 3 | 3 | Yes | Yes |
| PM_2.5_ | 2 | 100 | 4 | 3 | Yes | Yes |
| SO_2_ | 1 | 100 | 3 | 3 | Yes | Yes |

**References:**

Chen, C.-C., Wu, C.-F., Yu, H.-L., Chan, C.-C., Cheng, T.-J., 2012. Spatiotemporal modeling with temporal-invariant variogram subgroups to estimate fine particulate matter PM2.5 concentrations. Atmospheric Environment 54, 1-8.

Keller, J.P., Olives, C., Kim, S.Y., Sheppard, L., Sampson, P.D., Szpiro, A.A., Oron, A.P., Lindstrom, J., Vedal, S., Kaufman, J.D., 2015. A Unified Spatiotemporal Modeling Approach for Predicting Concentrations of Multiple Air Pollutants in the Multi-Ethnic Study of Atherosclerosis and Air Pollution. Environ Health Persp 123, 301-309.

Lindström J, Szpiro AA, Sampson PD, Bergen S, Oron AP. 2019. SpatioTemporal: Spatio- Temporal Model Estimation. R Package Version 1.1.9.1. Available: http://cran.r-project.org/web/ packages/SpatioTemporal/index.html [accessed 1 September 2020].

Tao Liu, J.X., Weilin Zeng, Jianxiong Hu, Xin Liu, Moran Dong, Jiaqi Wang, Donghua Wan, Wenjun Ma, 2019. A spatiotemporal land-use-regression model to assess individual level long-term exposure to ambient fine particulate matters. MethodsX 6, 2101-2105.

Wang, M., Keller, J.P., Adar, S.D., Kim, S.Y., Larson, T.V., Olives, C., Sampson, P.D., Sheppard, L., Szpiro, A.A., Vedal, S., Kaufman, J.D., 2015. Development of long-term spatiotemporal models for ambient ozone in six metropolitan regions of the United States: The MESA Air study. Atmos Environ 123, 79-87.

Wang, M., Sampson, P.D., Hu, J., Kleeman, M., Keller, J.P., Olives, C., Szpiro, A.A., Vedal, S., Kaufman, J.D., 2016. Combining Land-Use Regression and Chemical Transport Modeling in a Spatiotemporal Geostatistical Model for Ozone and PM2.5. Environ Sci Technol 50, 5111-5118.

Xu, J., Yang, W., Han, B., Wang, M., Wang, Z.S., Zhao, Z.P., Bai, Z.P., Vedal, S., 2019. An advanced spatio-temporal model for particulate matter and gaseous pollutants in Beijing, China. Atmos Environ 211, 120-127.


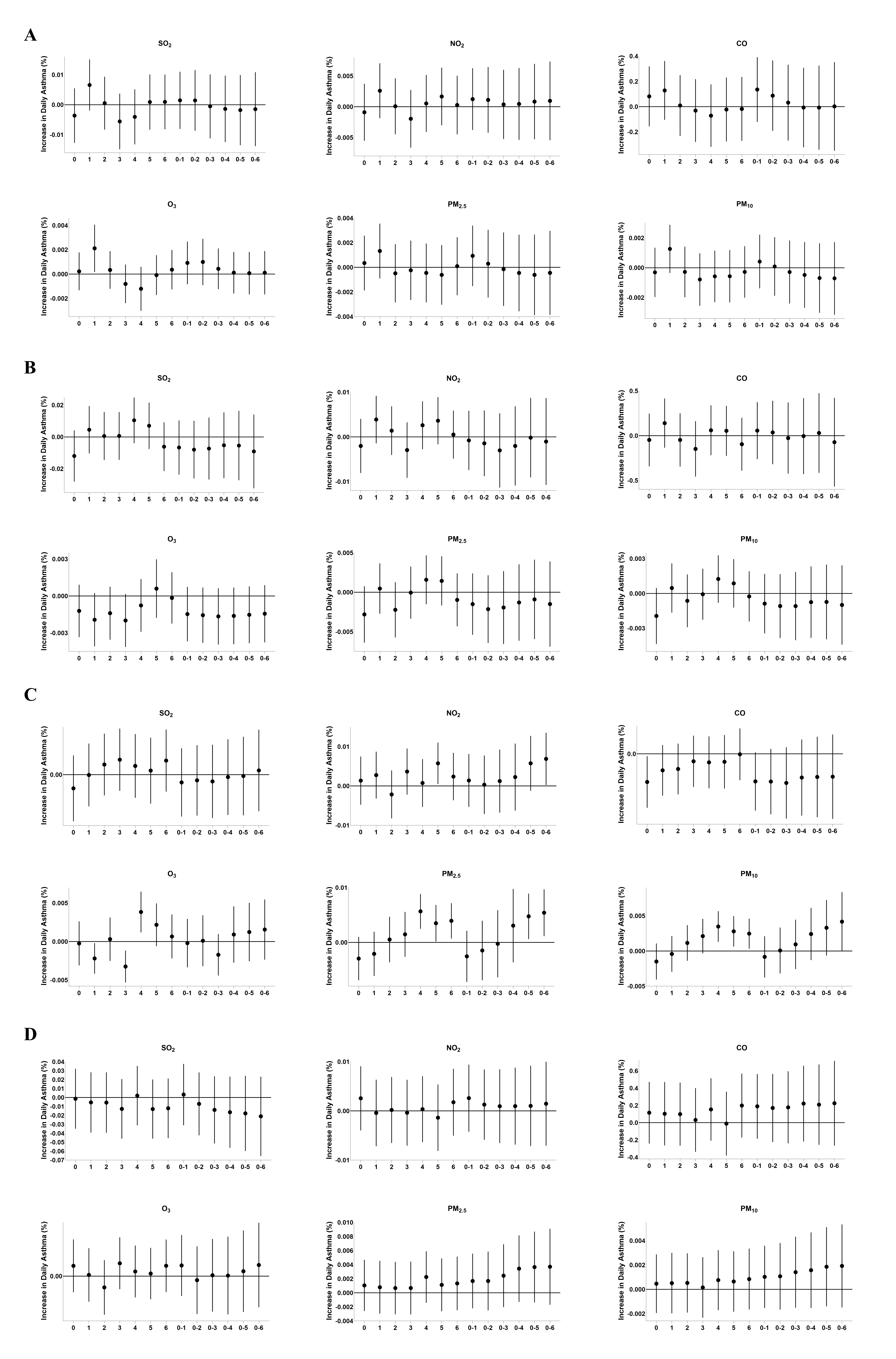


**Figure S1 The subjects were stratified by year to analyze the association.**

Percent changes (95% CI) in daily asthmatic hospitalization percentage deviations (%) stratified by gender. A: 2015; B: 2016; C: 2017; D: 2018.
